# Delay in the diagnosis of tuberculosis in Nepal

**DOI:** 10.1186/1471-2458-9-236

**Published:** 2009-07-14

**Authors:** Rajendra Basnet, Sven Gudmund Hinderaker, Don Enarson, Pushpa Malla, Odd Mørkve

**Affiliations:** 1 Centre for International Health, University of Bergen, Bergen, Norway; 2 International Nepal Fellowship, Nepalgunj, Nepal; 3 International Union against Tuberculosis and Lung Disease, Paris, France; 4 National Tuberculosis Programme, Kathmandu, Nepal

## Abstract

**Background:**

Identifying reasons for delay in diagnosis and treatment of tuberculosis is important for the health system to find ways to treat patients as early as possible, and hence reduce the suffering of patients and transmission of the disease. The objectives of this study was to assess the duration of delay in the diagnosis of tuberculosis and to investigate its determinants.

**Methods:**

A cross-sectional survey was conducted using a structured questionnaire in 307 new tuberculosis patients registered by the National Tuberculosis Programme (NTP) in all DOTS centres in Banke district of Nepal.

**Results:**

The median patient delay was 50 days, the median health system delay was 18 days, and the median total delay was 60 days. Sputum smear negative participants had significantly lower risk of patient delay. Smokers using >5 cigarettes per day had higher risk of patient delay and health system delay.

**Conclusion:**

Total delay in the diagnosis of tuberculosis in Banke district is shorter compared to other places in Nepal and neighbouring countries. The shorter delay for smear negative pulmonary tuberculosis raises suspicion that many of these patients are not examined according to the NTP manual before being diagnosed. Increasing public awareness of the disease and expansion of the facilities with assured quality could be helpful to reduce the delay in the diagnosis of tuberculosis.

## Background

More than 90% of the global tuberculosis cases and deaths occur in the developing world[1]. One third of the global burden of tuberculosis is from South East Asian Region where approximately 40% of total population has been infected with tuberculosis[2]. Nepal is a landlocked country between India and China with 31% of the total population of 26 million under the poverty line[3] and with 86% living in the rural area[4]. Tuberculosis is a major public health problem in Nepal. About 45% of the total population is infected with tuberculosis bacilli and the incidence of all forms of TB in Nepal is estimated to be 176/100000 population. The NTP introduced Direct Observed Treatment Short course (DOTS) strategy in 1996 and the number of deaths from tuberculosis is reduced since then, but still 5,000 to 7,000 patients die due to tuberculosis in Nepal every year[5]. There is clear political commitment to control tuberculosis, which has led to a case detection rate of sputum smear positive pulmonary tuberculosis of 64% (2006) and treatment success of 88% (2005)[6].

Delay in the diagnosis and treatment of tuberculosis cases spreads the infection in the community, increases severity of the disease and is associated with higher risk of mortality[7]. Knowing the delay in the diagnosis of TB is helpful for the health system to prevent spread of the disease. This study aimed to assess the duration of delay in the diagnosis of tuberculosis and to investigate its determinants. The findings of our study may be useful for further planning and policies in tuberculosis control in Nepal.

## Methods

The present study was approved by the Ethical Board of Nepal Health Research Council (NHRC). Informed consent was obtained from each participant. The study was conducted in Banke, a district in the Mid Western Region bordering in India in the south with population 447,000 in 2007. The district is divided into 46 Village Development Committees and one municipality. It is one of the relatively well developed districts of the country located on the flat Terai lowland. The public health sector has 49 facilities, 1 zonal hospital, 1 public health office, 3 primary health centres, 9 health posts and 35 sub-health posts; in addition there are 6 private sector facilities resulting in a total of 55 facilities providing DOTS services in Banke district, but only 48 of these had eligible participants during data collection. The reported notification rate of new pulmonary positive tuberculosis in Banke district was 92 per 100,000 population in 2007. We conducted a cross-sectional survey to assess retrospectively the duration of delay in the diagnosis of tuberculosis and its determinants. The study enrolled 307 new tuberculosis cases selected consecutively during their treatment period from the 55 DOTS centres/sub centres in the district. There was no tuberculosis patient on treatment at 3 sub-centres during the data collection period and responsible staff was on leave at 4 sub-centres during the training period for data collectors. Retreatment cases were not studied. A closed-ended questionnaire was administered to tuberculosis patients coming to collect their drugs at any stage of their treatment under National Tuberculosis Programme during June to July, 2007, by a trained staff member of DOTS treatment centres.

Using Epi-data 3.1 double data entry of the collected information was done and validated. Epi-data 3.1 was used for analysis of frequencies and tables, and SPSS 15.0 was used for logistic regression analysis. For the logistic regression modelling the outcome variables were "patient delay" (0 = 0–29 days delay, 1 = 30 days or more delay) and "health system delay" as more than 30 days (0 = 0–29 days delay, 1 = 30 days or more delay). Other cut-offs of delay were also tested but significant differences in delay by independent determinants were only found when 30 days was used as the cut-off. Unadjusted odds ratios (OR) were calculated. Age, sex, education, income, and distance are likely to influence delay and were among the covariates for adjusted odds ratio (AOR). All variables in the table were adjusted for in this model.

### Definitions

In this paper we used the following operational definitions: Patient delay is the time interval from the appearance of the first symptoms of tuberculosis until the first visit to any formal health care facility (health centres, hospitals or DOTS centres). Health system delay is the time interval from the first consultation at any formal health facility until the date of diagnosis. Total delay is the sum of patient delay and health system delay. A case of tuberculosis is a patient in whom tuberculosis has been confirmed by bacteriology or diagnosed by a clinician. A pulmonary case is a patient with tuberculosis disease involving the lung parenchyma. A case of sputum smear positive pulmonary tuberculosis is a patient with at least two initial positive sputum smears, or one sputum smear positive plus radiographic abnormalities consistent with active pulmonary tuberculosis as determined by a clinician; or one sputum specimen positive plus culture specimen positive for *Mycobacterium tuberculosis*. A sputum smear negative pulmonary tuberculosis case is a patient diagnosed with pulmonary tuberculosis by a clinician not meeting the above criteria for smear-positive disease. A case of extra pulmonary tuberculosis is a patient with tuberculosis of organs other than the lungs. A "new case" is a patient who has never had treatment for tuberculosis or who has taken anti-tuberculosis drugs for less than one month.

## Results

Table 1 shows the characteristics of the participants of this study. A total of 307 tuberculosis patients were included in our study. Among them, 139 (45.3%) participants had sputum smear positive pulmonary tuberculosis, 136 (44.3%) had sputum smear negative tuberculosis and 32 (10.4%) had extra pulmonary tuberculosis. Females comprised 41% of the participants. The majority of the participants were illiterate (62.2%), the main occupations of the participants were peasant (48.2%) and house wife (19.9%); and 76% of participants reported less than Nepali Rupees 2,500 (USD 36 per month ~USD 1 per household) family income per month. Smokers constituted 46.9% of participants and 28.7% of participants reported regular use of alcohol before diagnosis.

**Table 1:** Characteristics of Participants

**Characteristics**		**n**	**%**	**Median patient delay (d)**	**Median health system delay (d)**
Type of tuberculosis	Sputum smear positive	139	45.3	60	20
	Sputum smear negative	136	44.3	33	15
	Extra pulmonary	32	10.4	50	30
Age	≤ 14 years	32	10.4	42	20
	15 – 54 years	217	70.7	55	18
	≥ 55 years	58	18.9	60	19
Sex	Male	181	59.0	35	15
	Female	126	41.0	54	20
Marital status	Single	73	23.8	36	15
	Married	227	73.9	57	20
	Divorced/widow	7	2.3	-	-
Education level	Illiterate	191	62.2	57	20
	Literate	116	37.8	40	15
Occupation	Peasant	148	48.2	60	16
	House wife	61	19.9	45	20
	Others	98	31.9	37	20
Monthly family income	<NRs2,500	233	75.9	50	15
	≥ NRs2500	74	24.1	45	20
Smoking habit	No	163	53.1	36	15
	Yes	144	46.9	60	24
Alcohol consumption	No	219	71.3	42	18
	Yes	88	28.7	60	20

The median patient delay was 50 days; the median health system delay was 18 days; and the median total delay was 60 days. Patient delay represented 73% of total delay.

Table 2 shows the regression analysis. Sputum smear negative participants had significantly lower risk of patient delay (AOR 0.5, 95% CI 0.28–0.80). Smokers using more than 5 cigarettes per day had significantly higher risk of patient delay (AOR 2.7, 95% CI 1.39–5.38%) and the point estimates of risk of participants using 1–5 cigarettes per day were lower but increased, indicating a dose-response association. Smokers using more than 5 cigarettes per day had significantly higher risk of health system delay (AOR 2.4, 95% CI 1.18–4.79).

**Table 2:** Logistic regression analysis of risk factors for patient delay and health system delay

			**%**	**Patient delay (>30 days)**		**Health system delay (>30 days)**	
			
**Factors**		**N**		**OR (95% CI)**	**AOR (95% CI)**	**OR (95% CI)**	**AOR (95% CI)**
Type of tuberculosis	Sputum smear positive	139	45.4%	1.0	1.0	1.0	1.0
	Sputum smear negative	136	44.4%	0.5 (0.33–0.88)	0.5 (0.28–0.80)	0.8 (0.50–1.53)	0.9 (0.51–1.65)
	Extra pulmonary	31	10.1%	1.2 (0.53–2.93)	1.3 (0.53–3.08)	1.0 (0.41–2.40)	1.0 (0.41–2.58)
Age group	≤ 14 years	32	10.5%	1.0	1.0	1.0	1.0
	15 – 54 years	216	70.6%	0.5 (0.22–1.30)	1.0 (0.51–2.32)	1.2 (0.48–2.89)	1.1 (0.46–2.81)
	≥ 55 years	58	19.0%	0.6 (0.30–1.07)	1.8 (0.71–4.60)	1.0 (0.36–2.92)	0.9 (0.33–2.88)
Sex	Male	181	59.2%	1.0	1.0	1.0	1.0
	Female	125	40.8%	0.8 (0.53–1.36)	1.2 (0.73–1.98)	0.8 (0.45–1.33)	0.8 (0.44–1.37)
Marital status	Single	72	24.1%	1.0	1.0	1.0	1.0
	Married	227	75.9%	1.5 (0.87–2.55)	0.7 (0.35–1.40)	1.4 (0.73–2.72)	1.8 (0.76–4.24)
Education	Illiterate	190	62.1%	1.0	1.0	1.0	1.0
	Literate	116	37.9%	0.7 (0.42–1.10)	0.8 (0.46–1.26)	1.2 (0.74–2.14)	1.2 (0.67–2.12)
Occupation	Peasant	147	55.1%	1.0	1.0	1.0	1.0
	House wife	61	22.8%	0.7 (0.37–1.27)	0.5 (0.24–1.23)	0.7 (0.34–1.57)	0.6 (0.25–1.61)
	Others	59	22.1%	0.6 (0.31–1.05%)	0.7 (0.35–1.29)	1.5 (0.75–2.88)	1.5 (0.73–3.06)
Smoke	No	162	52.9%	1.0	1.0	1.0	1.0
	Yes 5 ≤ per day	54	17.6%	1.3 (0.73–2.32)	1.4 (0.76–2.65)	1.0 (0.52–2.10)	1.1 (0.52–2.29)
	Yes >5 per day	82	26.7%	2.4 (1.32–4.53)	2.7 (1.39–5.38)	2.1 (1.17–3.99)	2.4 (1.18–4.79)
Alcohol	No	219	71.3%	1.0	1.0	1.0	1.0
	Moderate	38	12.3%	1.1 (0.56–2.27)	1.2 (0.58–2.55)	1.4 (0.66–3.08)	0.7 (0.32–1.48)
	Heavy	48	15.6%	1.8 (0.90–3.50)	1.9 (0.91–3.91	1.4 (0.72–2.91)	0.9 (0.34–2.41)
Income	<NRs. 2,500	232	75.8%	1.0	1.0	1.0	1.0
	≥ NRs.2,500	74	24.2%	0.9 (0.52–1.52)	1.0 (0.59–1.77)	1.2 (0.64–2.25)	0.7 (0.40–1.47)
Travel time	≤ 1 hour	281	91.8%	1.0	1.0	1.0	
	>1 hour	25	8.2%	1.4 (0.60–3.47)	1.5 (0.60–3.59)	0.8 (0.28–2.13)	0.7 (0.25–1.97)

OR = Odds ratio, AOR = Adjusted odds ratio. All variables in the table were included as covariates in the logistic regression model.

The majority of the participants reported multiple symptoms of tuberculosis. Cough was the most frequently reported symptom (83%) followed by fever (74%) and weakness (64%). Most patients (58.3%) first sought advice from family members/relatives/friends for their symptoms. Government health facilities were contacted by 22.8% of the participants to seek their first advice, private practitioners/pharmacists/drug sellers by 10.4%, and traditional healers by 4.2%. Most of the participants (60.9%) reported that they walked to DOTS treatment centres, and 34.2% used bicycle. The median time from patients' residences to DOTS centres was 30 minutes. The majority of the participants (61.8%) consulted private practitioners/pharmacists for tuberculosis symptoms at least once before tuberculosis diagnosis. Traditional healers were visited by 16.3% of participants.

## Discussion

A major strength of this study is its focus on NTP and its designated diagnostic centres. This is where most of the cases in Nepal are diagnosed. But this is also a weakness, since it excludes a substantial and very interesting part of the tuberculosis burden treated in the private sector, and our study can say little about diagnostic delay in that setting. Another weakness is the dependence on patients' recall for all the data on delay. Self-reporting is not exact, it depends on recall. However, this is used in similar studies and hence comparison with other locations may still be valid. Still, recall bias must be kept in mind when interpreting the results. Digit preference as we observe in our data is typical for data from self-reporting.

In our study the median patient delay was 50 days. Shorter median patient delay was reported from Kathmandu valley (27 days)[8] and South India (20 days)[9], and longer patient delay was reported from Ethiopia (60 days)[10] and Tanzania (120 days)[11]. The differences could be because of variation in the definition of patient delay and selection of the study population. Our definition of patient delay is the time interval between the first symptom of tuberculosis and the first visit to "any formal health care facilities", whereas some references defined it as "any health facility". We have included sputum smear positive, sputum smear negative and extra pulmonary tuberculosis. Some studies included sputum smear positive pulmonary tuberculosis only and others included pulmonary tuberculosis and very few studies included extra pulmonary patients.

The median health system delay was 18 days. Longer health system delay was reported from Kathmandu valley (39 days)[8] and South India (23 days)[9], and shorter median health system delay was reported from Ethiopia (6 days)[10] and Tanzania (15 days)[11]. In our study, health system delay represents the time interval from the first visit to any formal health care facilities until the date of diagnosis. It does not include the period between the diagnosis of tuberculosis and the beginning of treatment, but this time from diagnosis to treatment initiation is fairly short in Nepal and has been estimated to be 2 days in Kathmandu valley[8] Health system delay is mainly due to health care facilities. It may be due to lack of diagnostic facilities, availability of trained staff, lack of quality of services and lack of effective supervision. It may also be due to patients not keeping their appointment for getting the examination results. We found shorter duration of health system delay compared to most of the other studies. It could be because the infrastructure of health services is relatively good in this district and with easy access to diagnostic centres and flat topography, or because there are not as many private health care providers as in Kathmandu valley.

Sputum smear negative pulmonary tuberculosis patients had significantly lower risk of patient delay in our study, which may sound surprising. Sputum smear positive pulmonary tuberculosis patients had shorter health system delay compared to smear negative in Ethiopia, Southern Taiwan and London[10,12,13]. The lower risk of patient delay for smear negative pulmonary tuberculosis in our study may be because they have early and less advanced disease or they may be diagnosed rapidly on very limited clinical impression only. Many of the doctors, particularly doctors in private sector, may not follow the NTP manual for diagnosis of tuberculosis, but may diagnose tuberculosis patients quickly and send them to NTP for free tuberculosis treatment. Part of the success of NTP in Nepal could be the policy of accepting a doctor's diagnosis without discussion, and rather encourage smear microscopy in all suspects.

Smokers using more than 5 cigarettes per day had significantly higher risk of patient delay, and the association seemed to be dependent on "dose" since participants using less smoke had less risk than heavy smokers (though not statistically significant) but increased risk compared to non-smokers (see Table 2). A study from New Zealand had similar findings[14]. Smokers may have cough every day and they do not recognize coughing as a symptom of disease and will therefore be delayed to seek health care until they have another annoying symptom or they are seriously ill. Smokers using >5 cigarettes per day also had higher risk of health system delay, and this may indicate that health care staff also may think the cough is due to smoking and do not think of tuberculosis as a differential diagnoses initially and rather focus on ill-defined symptoms.

Banke is a well developed district of Nepal, so the infrastructure of health care system is fairly good and DOTS centres are easily accessible for most patients of this district on the flat Terai plain. The median estimated time from patient home to DOTS centres by foot was 30 minutes. This time may be much longer in hilly areas of the country with much more strenuous travelling.

## Conclusion

In this study on delay in diagnosis of tuberculosis in Nepal we have shown that the median total delay is 60 days and most of it is patient delay (50 days). Smear positive tuberculosis patients have longer delay than smear negative patients, and smoking is a risk factor for both patient delay and health system delay.

## Competing interests

The authors declare that they have no competing interests.

## Authors' contributions

RB and SGH contributed to design and methods. RB collected the data. RB, SGH and DE analysed the data. RB, SGH, PM and DE all contributed to concept, writing and editing of the paper.

## Pre-publication history

The pre-publication history for this paper can be accessed here:


